# Anesthetic Approach for a Pediatric Patient With Facioscapulohumeral Muscular Dystrophy

**DOI:** 10.7759/cureus.69058

**Published:** 2024-09-10

**Authors:** Conceição C Santos, Carla I Ferreira, Erica Carvalho, Maria R Amaro, Cristina Gomes

**Affiliations:** 1 Anesthesiology and Perioperative Medicine, Hospital de Braga, Braga, PRT

**Keywords:** facioscapulohumeral muscular dystrophy, muscular dystrophies, neuromuscular nondepolarizing agents, pediatric anesthesia, sugammadex

## Abstract

Facioscapulohumeral muscular dystrophy (FSHD) belongs to the group of rare diseases known as muscular dystrophies. Patients with muscular dystrophies face a heightened risk of intraoperative complications, including severe hyperkalemia and acute rhabdomyolysis. This case report outlines the anesthetic approach employed for a pediatric patient diagnosed with FSHD undergoing a planned exploratory tympanotomy. To the best of our knowledge, it is the first documented case in the literature detailing pediatric general anesthesia in a patient with FSHD, with the additional use of neuromuscular blockade reversal with sugammadex.

## Introduction

Muscular dystrophies constitute a group of degenerative genetic disorders characterized by progressive muscle wasting and varying degrees of weakness [1]. These conditions can be categorized based on the pattern of muscle weakness and the underlying genetic defect. Additionally, if symptoms appear within the first six months of life, the dystrophy is classified as congenital [1,2]. Patients with muscular dystrophies are at high risk of intraoperative complications. General anesthesia may exacerbate respiratory and cardiovascular failure due to a marked sensitivity to several anesthetic drugs. Additionally, halogenated agents and depolarizing neuromuscular blocking agents can trigger life-threatening reactions, such as severe hyperkalemia and acute rhabdomyolysis, resembling malignant hyperthermia [2,3].

Facioscapulohumeral muscular dystrophy (FSHD) is a genetic muscular dystrophy caused by the misexpression of the DUX4 retrogene in skeletal muscles [4]. Despite its rarity, with a prevalence of one to nine per 100,000, it ranks as the third most prevalent form of muscular dystrophy in the general population [2]. FSHD has a variable age of onset, with symptoms typically appearing during adolescence and over 50% of individuals showing signs by age 20. Although the condition affects both genders, it has higher penetrance in males, who tend to experience symptoms earlier and with greater severity [5]. Phenotypes may vary, but generally, it is characterized by a progressive and asymmetric deterioration in muscle strength, often following a rostrocaudal pattern. This deterioration usually involves the face, periscapular region, and upper extremities, with relative sparing of the pelvic and lower limb muscles. Cardiac and respiratory involvement are rare, and life expectancy is nearly normal for most patients [6].

Currently, there is a lack of documented literature outlining the anesthetic and perioperative management of pediatric patients with FSHD. This case report seeks to bridge this gap, providing insights and suggesting strategies for enhancing the perioperative care of surgical patients with this dystrophy.

## Case presentation

A planned exploratory tympanotomy due to otosclerosis was proposed for a 12-year-old female American Society of Anesthesiologists (ASA) physical status II patient with a personal history of a genetic diagnosis of FSHD. The diagnosis was established during early infancy through genetic study, prompted by the paternal manifestation of the disorder. During the preoperative evaluation, the only symptom observed was mild conductive hearing loss, with no signs of neuromuscular abnormalities. The physical examination was otherwise normal, with vital signs showing a blood pressure of 116/77 mmHg, a heart rate of 78 bpm, and a peripheral oxygen saturation of 99%. The patient weighed 39 kg and measured 148 cm. There was no history of other medical conditions or prior surgeries. Preoperative laboratory assessments, including a complete blood count, electrolyte panel, coagulation study, and renal function analysis, revealed unremarkable findings (Table 1). General anesthesia was recommended for the patient. After explaining the risks and benefits to the legal representative, who understood and agreed to the plan, informed consent was provided and signed.

**Table 1:** Preoperative laboratory assessment Note: reference ranges are from the laboratories of the authors' institution.

Variable	Value	References ranges
Activated partial thromboplastin time (sec)	31.0	21.0-37.0
Chloride (mmol/L)	104	98-107
Creatinine (mg/dL)	0.7	0.42-0.71
Haemoglobin (g/dL)	14.2	11.5-15.5
Platelets (10^3^/uL)	322	180-400
Potassium (mmol/L)	4.2	3.5-5.1
Prothrombin time (sec)	13.6	8.0-14.0
Sodium (mmol/L)	138	136-145
White blood cells (10^3^/uL)	6.5	4.0-13.5

The anesthesia workstation was meticulously prepared the night before the surgery. Vaporizers were removed, and both the carbon dioxide absorbent and the anesthetic breathing circuit were replaced. The entire system was flushed with a fresh gas flow of oxygen at 10L/min for 10 minutes. On the day of the surgery, the patient was the first to enter the operating room. These precautions were implemented due to the hypothetical risk of anesthesia-induced rhabdomyolysis (AIR). Two peripheral venous accesses (22G and 18G) were established on each one of the upper limbs. Comprehensive monitoring included ASA standards I and II with temperature assessment and anesthetic depth with Bispectral Index™ (BIS™, Medtronic, Dublin, Ireland) and neuromuscular monitoring using a train-of-four (TOF) ratio with TOF-Watch® (Organon & Co., New Jersey, USA) while stimulating the ulnar nerve.

We performed total intravenous anesthesia using target-controlled infusion with propofol and remifentanil. At induction, propofol was administered using the Paedfusor model with an effect-site concentration of 3.5 mcg/mL, while remifentanil was administered through the Minto model with an effect-site concentration of 2 mcg/mL. Upon the loss of consciousness, a dose of 25 mg of rocuronium was given. After approximately three minutes, no responses were observed on the TOF monitor, prompting the uneventful intubation of the patient with an orotracheal tube following a laryngoscopy.

The effect-site concentration of propofol and remifentanil during maintenance of anesthesia was adjusted according to the anesthetic depth and hemodynamic values, respectively. Given the minimal blood loss and exposure, maintenance fluid therapy was administered using a polyelectrolyte solution, calculated according to the Holliday-Segar equation. To prevent nausea and vomiting, 4 mg of dexamethasone and 4 mg of ondansetron were administered intravenously, as were 800 mg of paracetamol and 20 mg of ketorolac for postoperative analgesia. The surgical procedure lasted two hours and 20 minutes, with uneventful anesthesia and surgery. Normothermia and normocapnia were diligently maintained throughout the procedure. No additional dose of rocuronium was administered. At the end of the surgery, we assessed the TOF ratio, which showed three out of four responses. The patient was reversed with 80 mg of sugammadex, and after confirming a TOF ratio above 90%, the extubation was successfully performed.

The post-anesthesia care unit stay was satisfactory during the immediate postoperative follow-up. Due to the potential risk of late complications, the patient was closely monitored in the pediatric intensive care unit for the initial 24 postoperative hours. The patient made a successful recovery with no signs of hyperkalemia or rhabdomyolysis. She was discharged from the hospital on the third postoperative day without any adverse events.

## Discussion

Anesthesia for a child with a muscle disease is always challenging because of the risk of perioperative complications. Information available on the anesthetic and perioperative handling of a child with FSHD is very limited.

The present case describes a pediatric patient with a genetic diagnosis of FSHD scheduled for a programmed exploratory tympanotomy. To optimize the outcome and minimize the risk of complications, several measures were implemented. Although there are not any reports of AIR with dystrophies other than Duchenne or Becker muscular dystrophy, the hypothetical risk of AIR on exposure to volatile anesthetics cannot be excluded [2]. Therefore, we chose to conduct an exhaustive preparation of the anesthesia workstation, giving priority to the patient as the first case of the day in the operating room. Total intravenous anesthesia was selected due to its established safety in neuromuscular disorders and the imperative to maintain hemodynamic stability, particularly blood pressure, during the surgical procedure that the patient was undergoing [2,7].

Patients with neuromuscular disease exhibit an increased sensitivity to nondepolarizing neuromuscular blocking agents; consequently, vigilant monitoring of the neuromuscular blockage is essential [2,8]. A neuromuscular blocking agent was used to prevent sudden patient movements because of the meticulous nature of the surgery. We opted to use rocuronium due to its effective reversal with sugammadex. Despite the existing recommendations for sugammadex use in neuromuscular disorders, our exploration revealed only one case report detailing its application in FSHD [9]. Notably, in our patient, both the administration of rocuronium and sugammadex were found to be safe, with no observed side effects.

The surveillance of these patients is crucial during the perioperative period for individuals with muscular dystrophies. Intraoperatively, active monitoring of expired carbon dioxide, electrocardiogram, temperature, and neuromuscular blockade allows for the identification of complications such as rhabdomyolysis, hyperkalemia, and alterations in sensitivity to neuromuscular blocking agents [2,10]. In the postoperative period, following recommendations, the patient remained under 24-hour monitoring in the pediatric intensive care unit with electrocardiogram and pulse oximetry to identify any decrease in cardiopulmonary functioning, as well as surveillance for signs of ongoing rhabdomyolysis [2,8].

The importance of the pre-anesthetic diagnosis of muscular dystrophy through genetic testing, conducted prior to the challenges associated with the induction of general anesthesia, is noteworthy [11]. Without such a preemptive study, the implementation of preventive measures, especially in anticipating and preventing anesthetic complications, would not have been possible.

## Conclusions

Facioscapulohumeral dystrophy is a rare condition with few documented anesthetic cases, none of which involve pediatric patients. Prior awareness of the genetic mutation enabled careful preparation and the selection of the optimal anesthetic approach, thereby minimizing the risk of major anesthetic complications. This case demonstrates a safe anesthetic approach for children with the genetic mutation for FSHD, incorporating the use of rocuronium and the reversal of neuromuscular blockade with sugammadex.

## References

[1] Emery AE (2002). The muscular dystrophies. Lancet.

[2] van den Bersselaar LR, Heytens L, Silva HC (2022). European neuromuscular centre consensus statement on anaesthesia in patients with neuromuscular disorders. Eur J Neurol.

[3] Gurnaney H, Brown A, Litman RS (2009). Malignant hyperthermia and muscular dystrophies. Anesth Analg.

[4] Yao Z, Snider L, Balog J, Lemmers RJ, Van Der Maarel SM, Tawil R, Tapscott SJ (2014). DUX4-induced gene expression is the major molecular signature in FSHD skeletal muscle. Hum Mol Genet.

[5] Preston MK, Tawil R, Wang LH (1993). Facioscapulohumeral muscular dystrophy. GeneReviews® [Internet].

[6] Wicklund MP (2013). The muscular dystrophies. Continuum (Minneap Minn).

[7] Trevisan CP, Accorsi A, Morandi LO (2013). Undiagnosed myopathy before surgery and safe anaesthesia table. Acta Myol.

[8] Radkowski P, Suren L, Podhorodecka K, Harikumar S, Jamrozik N (2023). A review on the anesthetic management of patients with neuromuscular diseases. Anesth Pain Med.

[9] Hélaine L, Le Cocq C, Saadi H, Abdelkrim N, Atti A (2014). Rocuronium and sugammadex use for the management of neuromuscular blockade in urgent abdominal surgery in a patient with Landouzy-Dejerine myopathy (Article in French). Ann Fr Anesth Reanim.

[10] van den Bersselaar LR, Snoeck MM, Gubbels M, Riazi S, Kamsteeg EJ, Jungbluth H, Voermans NC (2020). Anaesthesia and neuromuscular disorders: what a neurologist needs to know. Pract Neurol.

[11] Body SC (2009). Genomics: implications for anesthesia, perioperative care and outcomes. Adv Anesth.

